# Lattice-based digital signatures

**DOI:** 10.1093/nsr/nwab077

**Published:** 2021-05-08

**Authors:** Vadim Lyubashevsky

**Affiliations:** Zurich Research Laboratory, IBM Research, Switzerland

**Keywords:** lattice cryptography, digital signatures, standardization

Digital signatures and key exchange protocols are the two most important public key cryptographic primitives used in the electronic transmission of data. The goal of key exchange is to preserve the secrecy of the communication, while the goal of digital signatures is to guarantee the authenticity of the exchanged messages. Constructions of digital signature schemes based on classical mathematical assumptions appeared shortly following the invention of public key cryptography in the late 1970s. And just like with key exchange, the most efficient variants are based on number-theoretic problems that are believed to be (sub)-exponentially hard for classical machines, but are solved in polynomial time by Shor’s algorithm on a powerful-enough quantum computer. Also, like for key exchange, the most efficient constructions that we believe to be quantum safe are based on the presumed hardness of lattice problems over polynomial rings.

One interesting difference between key exchange and digital signatures is that key exchange appears to inherently require that some mathematical problem be computationally hard. Digital signatures, on the other hand, can be generically constructed from any one-way function [1,2]. So even though they certainly fall into the category of public key primitives based on their usage, their existence requires much weaker assumptions. Additionally, the transformation from a one-way function to a digital signature is not too inefficient. For example, the total parameter size (public key + signature) of the SPHINCS+ scheme [3] is around 40 kB. While these sizes are larger, and signing times considerably longer, than those of signatures based on factoring or discrete log, it is still a usable scheme for many applications. And being only based on symmetric assumptions (e.g. one wayness and collision resistance of cryptographic hash functions), its security is very attractive.

In order to be considered an interesting alternative to the above-mentioned signature, a scheme based on a mathematical assumption would need to have significant performance advantages. Schemes based on factoring and the discrete logarithm problem were significantly shorter and faster, and so the generic approach lay dormant for over four decades. The new quantum-safe schemes will need to have similar performance advantages if they are to be used in lieu of this safe approach. Below, we describe two techniques for constructing lattice-based digital signatures with output sizes being just a few kilobytes.

On a very high level, lattice-based signature constructions follow the two known approaches for constructing classical signatures. In the first approach, the signer outputs a function *f* and an image *y* = *f*(*x*) as his public key and keeps *x* as his secret key. To sign a message μ, he gives a non-interactive zero-knowledge proof that he knows an *x* satisfying *y* = *f*(*x*), using the message μ to create the ‘challenge’ *H*(μ) for the proof (where *H* is a public function that maps μ to something ‘random looking’). If the function *f* is one way then the verifier should be convinced that the proof could have only been created by the entity who knows *x*. A classic example of this type of scheme is the Schnorr signature scheme [4] based on the hardness of the discrete logarithm problem. The second approach is to create a function *f* together with a trapdoor *f*^−1^, output *f* as the public key and keep *f*^−1^ as the secret key. A message μ is signed by using the secret trapdoor to create a pre-image *x* such that *f*(*x*) = *H*(μ). Again, if the function *f* is one way then only someone in possession of a trapdoor should be able to invert it. An example of such a construction based on a ‘factoring-like’ assumption is the RSA signature scheme [5].

The high-level ideas for lattice-based signatures follow the above blueprints, but the technical details are significantly more involved. The main reason for the complications is the different algebraic structure of the hard one-way function underlying lattice cryptography. While the domain of the function in discrete log and RSA-based one-way functions are groups, the domains in lattice-based signatures are sets that are not closed under any operation—in particular, they are elements in a group that have small norms. This crucial small norm requirement precludes us from using uniformly random masking as in Schnorr signatures or having a trapdoor for a bijective one-way function as in RSA signatures. These barriers have, nevertheless, been overcome and the resulting digital signatures are quite practical.

In 2017, the US National Institute of Standards and Technology (NIST) began a ‘competition’ for a quantum-safe key exchange and digital signatures standard. At the time of this writing, this process is in the third round and there are two lattice-based signatures remaining—each following one of the above high-level designs. The CRYSTALS-Dilithium [6] scheme follows the Schnorr framework, but adds a crucial rejection-sampling step to keep the size of the coefficients small. The FALCON scheme [7] utilizes a randomized trapdoor sampling technique that uses a secret trapdoor for *f*^−1^ to produce *random* pre-images from a particular distribution. Because there is no longer a bijection, it is crucial to also have the property that the distribution of the outputted pre-images does not leak information about the trapdoor. Both schemes are relatively fast and their parameters (public key + signature size) are the shortest of all quantum-safe signature schemes.

While both schemes are based on lattices, they have rather different characteristics. FALCON has very short parameters (see Table 1), but entails a rather complicated procedure for signature generation. In particular, it uses (an optimized version of) the GPV sampler [8], which requires floating-point arithmetic with approximately 64 bits of precision. Requiring such high precision means that subtle implementation errors may not get detected even with rigorous testing. Dilithium, on the other hand, has larger parameters but a very simple implementation where all the sampling in the signing is done in a power-of-2 range; it is thus much less prone to implementation errors. It is quite possible that in the future both schemes will be used for different applications.

**Table 1.** Approximate parameter sizes (in bytes) for the CRYSTALS-Dilithium and FALCON digital signature schemes at approximately 128-bit security levels.

	CRYSTALS-Dilithium	FALCON
Public key	1300	900
Signature	2400	650
